# Serious game as oral histology learning strategy for undergraduate dental students; crossover randomized controlled trial

**DOI:** 10.1186/s12903-023-03286-3

**Published:** 2023-08-23

**Authors:** Lisa R. Amir, Irene C. Leonardy, Salsabila N. Dewatmoko, Rezon Yanuar, Dewi F. Suniarti, Erik Idrus, Kawin Sipiyaruk, Ria Puspitawati

**Affiliations:** 1 Department of Oral Biology, Faculty of Dentistry, Universitas Indonesia, Salemba Raya No. 4, Jakarta Pusat 10430, Jakarta, Indonesia; 2 Dental Education Unit, Faculty of Dentistry, Universitas Indonesia, Jakarta, Indonesia; 3 Dentistry Study Program, Faculty of Dentistry, Universitas Indonesia, Jakarta, Indonesia; 4 Department of Pharmacology, School of Dentistry, Health Sciences University of Hokkaido, Ishikari- Tobetsu, Hokkaido, Japan; 5 Department of Orthodontics, Faculty of Dentistry, Mahidol University, Bangkok, Thailand

**Keywords:** Serious Games, Oral histology, Undergraduate students, Innovative learning

## Abstract

**Background:**

Oral histology is perceived by dental students as a challenging subject and often struggle to recognize the long-term relevance of understanding the cells and tissues at the microscopic level. Serious games have been reported to have a positive effect on student cognitive skills and learning motivation. However, there is still a limited amount of research supporting the effectiveness of serious games as a learning method in dentistry. The present study aimed to evaluate the impact of serious game of *HistoRM* as a complementary learning strategy for oral histology.

**Methods:**

The study design was a crossover randomized controlled trial. A total of 74 first year dental students of Universitas Indonesia participated in the study and divided into 2 groups. Study intervention included *HistoRM* game for 3 days followed by a combination of *HistoRM* and script-based handouts for another 4 days. The groups represented different intervention sequences. Evaluation was performed using pre-test, post-test on day 3 and 7 and a questionnaire.

**Results:**

The data showed significant improvement of student cognitive skills (p < 0.001) and it was influenced by the number of game missions completed. Students who completed the whole 15 missions have a higher day-7 post-tests scores (p = 0.03). Perception of dental students on *HistoRM* was positive in all domains tested, the learning content, games and learning experience domains. Immediate feedback given after each gameplay helped the students understand the subject matters.

**Conclusion:**

Serious game of *HistoRM* effectively improved students’ understanding of oral histology learning outcome and provided more interesting learning experiences. This innovative learning can be recommended as a complementary learning strategy of oral histology for dental students.

**Supplementary Information:**

The online version contains supplementary material available at 10.1186/s12903-023-03286-3.

## Background

Oral histology course, study the science of the microscopic structure of cells and tissues, is an integral part of the dental curriculum. Previously, it was reported that dental students find oral histology as a difficult subject [1, 2]. The lack of understanding of the importance of recognizing the complexity of tissue organization and its function to support the learning and understanding the pathogenesis of diseases might contribute to this perception [1–6]. Lesson materials may be partially or entirely forgotten by students when a large amount of complex study materials is provided for students to learn in a short period of time [7]. Moreover, student disinterest may play a role in weak performances in higher education [8]. Conventionally, the didactic learning approach of oral histology materials is delivered as knowledge transfer through classroom lectures and practical demonstrations of slides under the microscope. The approach does not stimulate students’ interactive discussion in the classroom nor active engagement. As the current medical and dental students are of the digital natives generation, they gravitated toward modern electronic learning tools that provide fast and efficient feedback [8]. Clearly, there is a need for a new learning approach that could engage students in the learning process, emphasize the learning outcomes and the relevance of oral histology in dentistry. An interactive learning environment that could create an interesting learning atmosphere without neglecting the learning materials, will increase student’s knowledge, skills, and motivation, encouraging student engagement, and at the same time increasing learning and solving problems.

Contemporary educational technology is implemented in recent years to scaffold knowledge transfer to the learners in a situated and multimodal approach [9]. Gamification refers to the application of game theory, techniques and mechanics outside the context of traditional game activities [10]. In the past several years, trends in educational research show an increasing interest in incorporating games in learning approaches. Game-based learning refers to a strategy to enhance knowledge and skill acquisition, through game content and game play, where game activities involve problem solving and challenges that provide players/learners with a sense of achievement of the defined learning outcomes [11]. Games are called serious when they have a pedagogical purpose [12]. The introduction of games in education has been demonstrated to stimulate learning and improve learner satisfaction. Previous reviews on serious games in healthcare education indicate the most frequent outcome investigated was the improvement in knowledge acquisition and is perceived as a more engaging learning approach compared to traditional methods [10–17]. Although serious games are not considered mainstream material in medical and dental teaching, it provides tools to enhance interactivity in healthcare education including dental education. Serious games could overcome limitations of asynchronous learning, the learning strategy that became widely used during this frequent pandemic time [15].

The evaluation of undergraduate dental curriculum in the Universitas Indonesia demonstrated a similar phenomenon with others [1–6]. Student perception towards their level of understanding of oral histology was still unsatisfactory. In particular, their understanding of the long-term relevance of oral histology, which includes the complexity of functional networks and pathological processes. The implementation of innovative learning strategies such as serious games to enhance knowledge acquisition, motivation and engaging learning environment can therefore be a solution. However, research on the effectiveness and use of serious games as a complementary learning method in dentistry has not been well-studied. The present study was aimed to evaluate the effectiveness of the implementation of a *HistoRM* serious game specifically designed as a complementary learning of oral histology for undergraduate dental students.

## Methods

### Study design

**Fig. 1:**
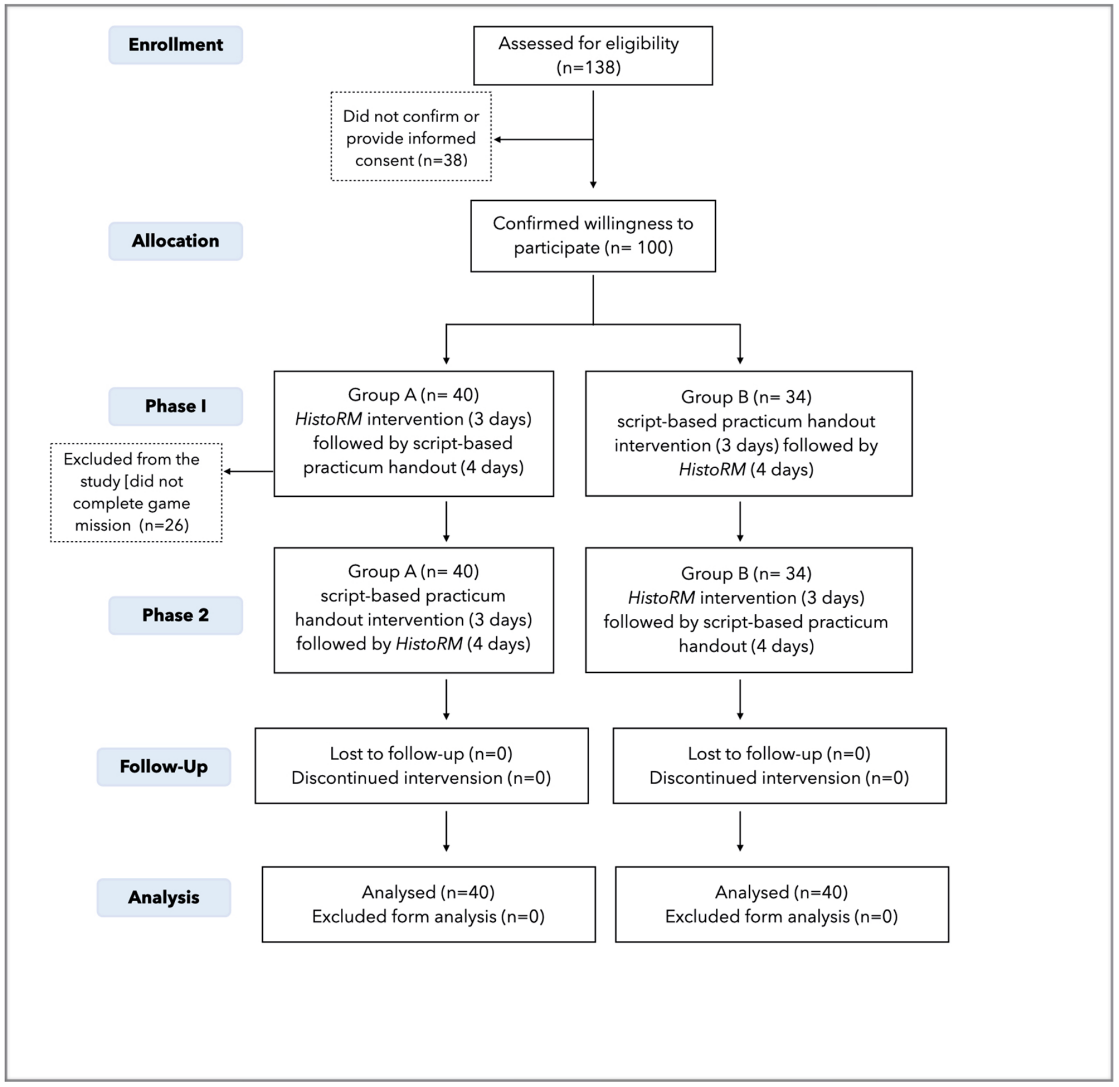
CONSORT Flow Diagram of the Randomized Controlled Trial

This research was conducted in August 2022 - January 2023, using a crossover randomized controlled trial, two-arm RCT with 1:1 allocation [18, 19]. Ethical approval was obtained from Dental Research Ethics Committee Universitas Indonesia (87/ethical approval/FKGUI/X/2022). The trial was registered to ISRCTN Registry Service (23.03.2023), registration number ISRCTN11006820. The results of the study were reported using the Consolidated Standards of Reporting Trials (CONSORT) Guidelines (Fig. 1) [20].

### Study participants

**Fig. 2:**
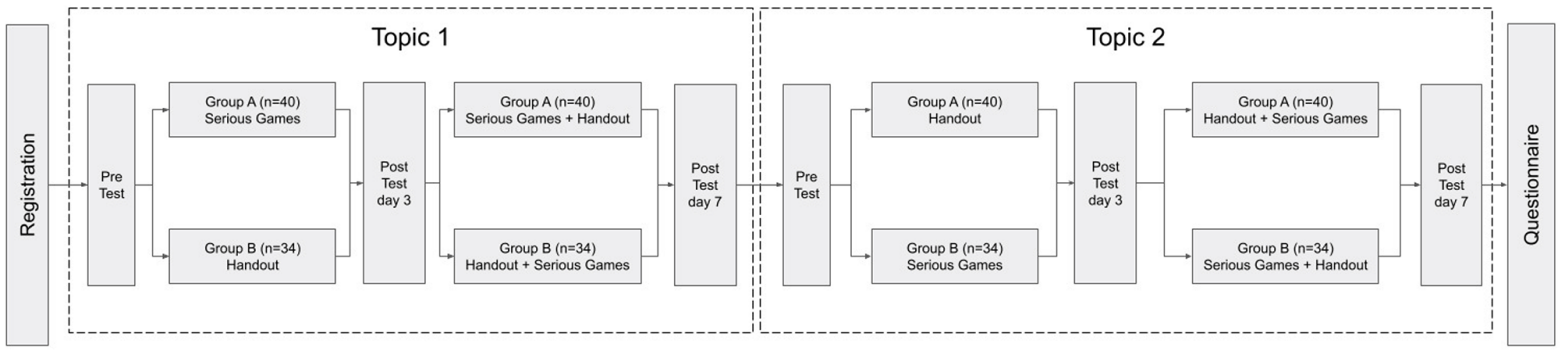
Research timeline

Study participants were undergraduate 1st year dental students who studied the basic oral biology practicum course in the 1st semester of the 2022/2023 academic year. Their participation was voluntary-based and third year dental students (ICL and SND) were involved in the recruitment of study participants. All respondents filled out and submitted the informed consent form. G*power statistical software was used to calculate sample size [21]. We estimated sample size of 45 participants in each group would be sufficient to detect statistical significant with a power of 0.8, significant level of 0.05, assuming an effect size of 0.6. Students were randomly divided into two groups. Prior to the intervention, the GPA score, pre-test knowledge score and game preference in the two groups were evaluated to minimize bias. It was revealed that there were no significant difference of the scores nor the preference between the two groups. Pre-tests were given before the study started to evaluate their prior knowledge. Two learning materials were prepared with complementary learning of *HistoRM* game, topic 1 was the histology of oral mucosa, and topic 2 was the histology of tooth and its supporting structure. The intervention consisted of the *HistoRM* game and the practicum handouts (script-based). Group A played *HistoRM* game of topic 1 for three days, and were later given the practicum handouts, while group B received the practicum handouts of topic 1 in the first three days, and played *HistoRM* game between day 3–7. After three days, students in group A and B could access both interventions. Post-tests evaluation was carried out on day 3 and day 7 (Table 1). The experimental study was repeated for topic 2. In the first three days, group A received the practicum handouts while group B played HistoRM game, implementing the crossover experimental design [18, 19]. At the end of study, participants were asked to fill out a survey to evaluate their understanding level of the learning material and their perception of learning experiences (Fig. 2).

**Table 1:** Description of Intervention Groups

**Topic 1 (Microscopic Structure of Oral Mucosa)**		
Post-test	Intervention	
	Group A	Group B
Day 3	Game only	Script-based Hand-out only
Day 7	Game (3 days) + Script-based Hand-out (4 days)	Script-based Hand-out (3 days) + Game (4 days)
**Topic 2 (Microscopic Structure of Tooth and Its Supporting Structures)**		
Post-test	Intervention	
	Group A	Group B
Day 3	Script-based Hand-out only	Game only
Day 7	Game (3 days) + Script-based Hand-out (4 days)	Script-based Hand-out (3 days) + Game (4 days)

### Serious game

**Fig. 3:**
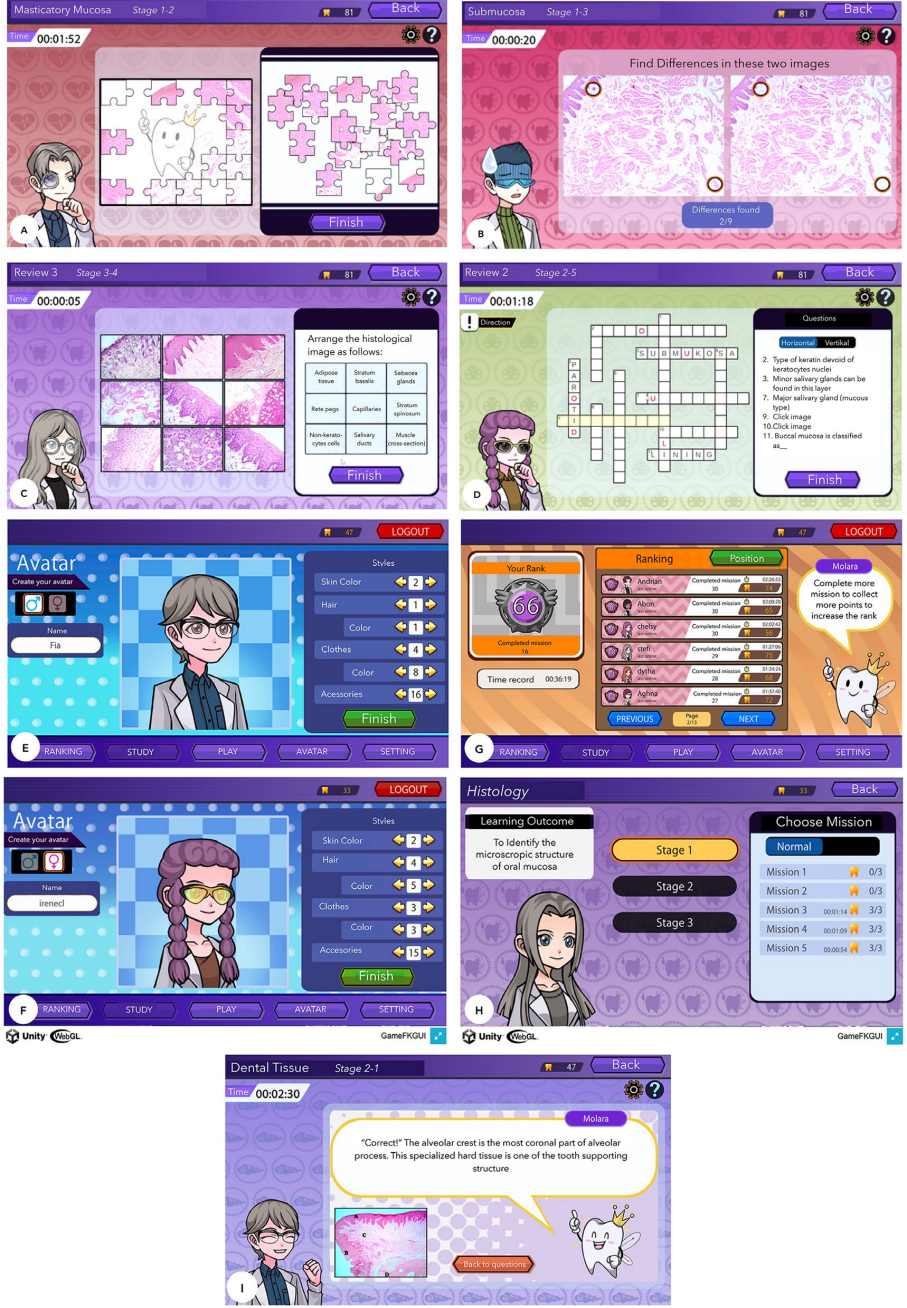
*HistoRM* Games. The gameplay included jigsaw puzzle (**A**); find the differences (**B**); match the pictures (**C**); and crosswords (**D**); Custom-made avatar design was created as entertainment elements of the games (**E-F**); Leaderboard showed student achievement and number of rewards (**G-H**) Molara character was created to give game instruction, hints and feedback (**I**)

Serious game called *HistoRM* was developed with Unity game platform and was integrated in the SCORM activity in the Universitas Indonesia moodle-based learning management system (EMAS). This education puzzle-based game consisted of four gameplays: (1) jigsaw puzzle; (2) find the differences; (3) match the pictures; and (4) crosswords (Fig. 3A-D). Two topics of oral histology learning subjects were used in *HistoRM* game consisted of: (1) Histology of oral mucosa, and (2) histology of tooth and its supporting structure. Each topic was divided into three stage levels, and each stage contained five missions, a total of 15 missions for each topic (Fig. 3E). Mission can be divided into two sessions (puzzle game session and quiz session). Students were required to clear the puzzle game session first before continuing to the quiz session, and were able to repeat the mission multiple times if they wanted to relearn the subject matter. One-to three-star points can be collected in every mission, depending on the winning condition such as the time to complete the mission, the number of mistakes, and the number of hint-feature used. These star points were collected and determined the player ranking in comparison to other players and were monitored in the leaderboard ranking system (Fig. 3F). There was no time limit in playing *HistoRM* game.

We incorporated the fun elements into the *HistoRM* game which included the custom-made avatars that represented the players, custom-made background and music. Players can design the avatars (hairstyle, hair color, clothes and other accessories) (Fig. 3G-H). Facial expression of the avatar changes based on the process when they played the games. A character known as Molara, a human-tooth form creature was developed and served to guide the students. In the beginning, Molara gave the explanation of learning outcomes of each stage, and gave instruction on how to play the puzzle-based game. During the puzzle game session, Molara offered the hint-feature when the players had difficulties to complete the mission. At the end of the quiz session, Molara provided immediate feedback and explanation related to the quiz topics (Fig. 3I).

### Assessments

Pre-test was carried out to determine student’s prior knowledge before the intervention. The impact of *HistoRM* as complementary learning was assessed with post-tests. Post-tests were given at day 3, to compare student’s cognitive skills in *HistoRM* group with practicum handouts (script-based) group, while post-test was carried out at day 7 to assess students’ cognitive skills between the groups tested which have been given both interventions in a different order. Pre- and post-test consisted of 30 multiple-choice questions that were shuffled between and within questions, and were carried out in EMAS.

### Questionnaire

The questionnaire consists of 21 questions in which 14 questions were answered by a 4-point Likert scale. Questions were divided into general information (7) and 3 domains consisted of learning content domain (3), game aspect domain (5), and learning experience domain (6). Fifteen students were selected for validity and reliability test of the questionnaire. The validity and reliability of the questionnaire were tested using face validity, Cronbach alpha, and the ICC test. The total Cronbach alpha score was 0.85 and can be categorized as reliable. The reliability of the questionnaire evaluated from the Intraclass Correlation Coefficient score had an excellent agreement (r = 0.98, 95% Confidence Interval (CI), 0.94–0.99).

### Statistical analysis

Descriptive statistics were computed and data normality was tested with Shapiro-Wilk. Bivariate analyses were performed using the SPSS 25.0 version. The level of statistical significance was accepted at 0.05.

## Results

### General information

Initially, there were 100 (73%) out of 137 undergraduate 1st year dental students from regular and international class who filled out the informed consent for their participation in the study. Students were randomly assigned into 2 groups (A and B). During the trial, a total of twenty-six students did not complete a minimum of 10 missions per topic within the designated time frame, and were excluded from the analyses. The reasons for students not being able to complete the required missions were high study load and *HistoRM* game could be played only on a laptop/desktop. Therefore, seventy-four students (53.6% of all first-year students) were included in the analysis.

Before the intervention, students were given pre-tests, and the scores revealed no statistically significant difference of student cognitive skills in group A and B for both oral histology topics (Table 2). A total of 59 (79.5%) participants like to play games in their spare time, and 54 students (72.4%) were not familiar with serious games prior to the trial (Table 1). The frequencies of students playing *HistoRM* game were varied, 42 (56.7%) of students played *HistoRM* game more than 2 times during the study, followed by 2 times (31 students, 42%) and 1 time (1 student, 1.3%). The average duration of play in the *HistoRM* game was 85 ± 25.7 min. The most liked puzzle-based gameplay types were jigsaw puzzles (58.1%), followed by find the difference (37.8%) and match the picture (2.7%). Crosswords games had the least favored gameplay (1.4%).

**Table 2:** General Information of Study Participants

		Topic 1		Topic 2	
		Group A (n = 40)	Group B (n = 34)	Group A (n = 40)	Group B (n = 34)
Gender					
	Male	8 (20%)	3 (8.8%)	8 (20%)	3 (8.8%)
	Female	32 (80%)	31 (91.2%)	32 (80%)	31 (91.2%)
Age in years (mean ± SD)		18.1 ± 0.6	17.9 ± 0.6	17.9 ± 0.6	18.1 ± 0.6
Do you like playing games?					
	Yes	33 (82.5%)	26 (76.5%)	33 (82.5%)	26 (76.5%)
	No	7 (17.5%)	8 (23.5%)	7 (17.5%)	8 (23.5%)
How often do you play games in a week? (in hours)					
	0–9 h	36 (90%)	33 (97.1%)	36 (90%)	33 (97.1%)
	10–19 h	4 (10%)	1 (2.9%)	4 (10%)	1 (2.9%)
	>20 h	-	-	-	-
Have you previously known what serious games are?					
	Yes	15 (37.5%)	6 (17.6%)	15 (37.5%)	6 (17.6%)
	No	25 (62.5%)	28 (82.4%)	25 (62.5%)	28 (82.4%)
Pre-test score (mean ± SD)		54.25 ± 14.20	51.18 ± 12.17	50.67 ± 13.78	45.88 ± 12.34

### The impact of HistoRM Game on cognitive skills

**Fig. 4:**
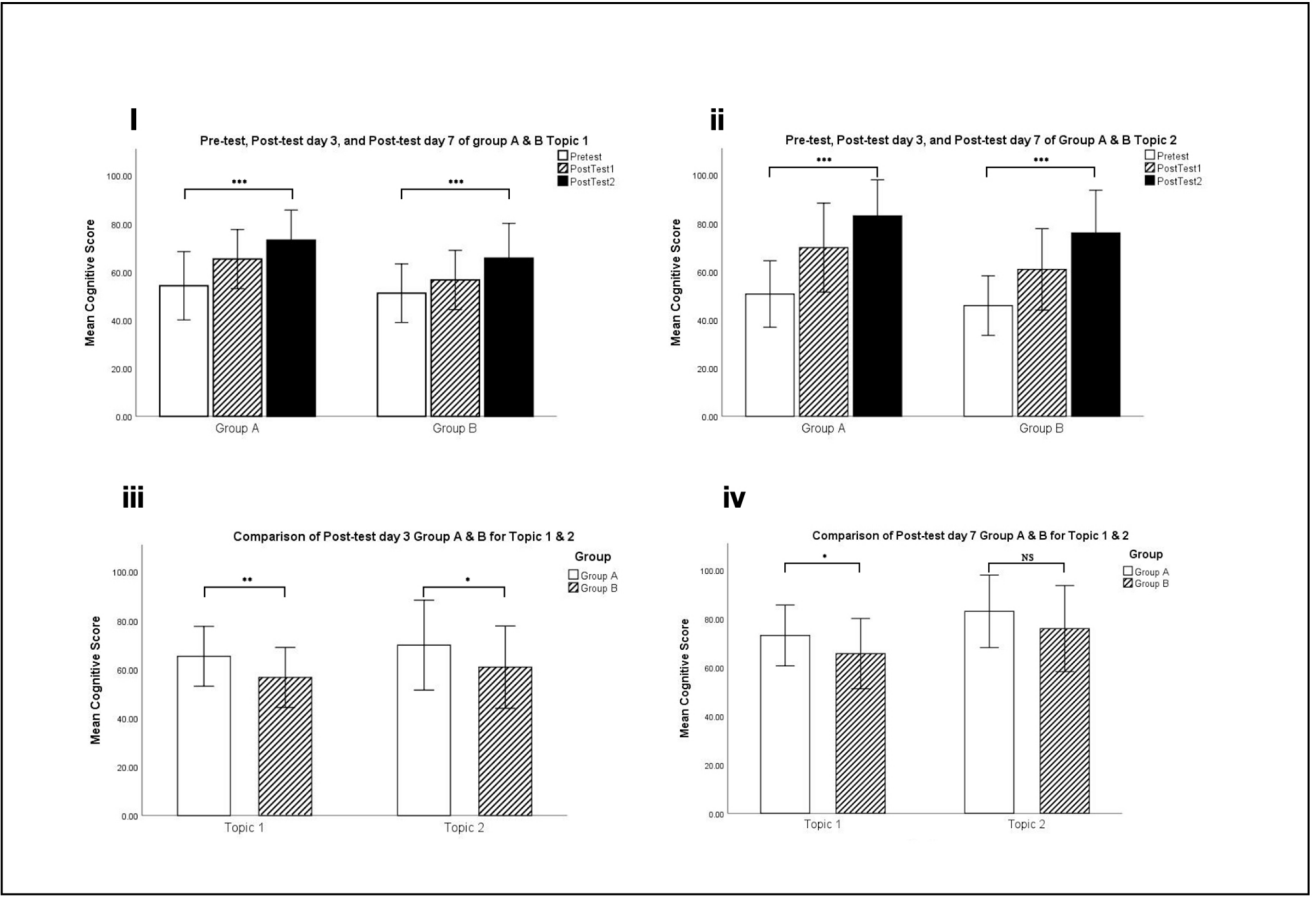
Results of student cognitive skill scores. Significant increased of cognitive skills scores were observed pre- and post tests in topic 1 (i) and topic 2 (ii); The order of intervention influenced the scores at day 3 (iii); but no significant different in the two groups tested at day 7 for topic 2 (iv)

*HistoRM* game interventions were carried out for two topics of oral histology. In topic 1 (histology of oral mucosa), the significant increased of cognitive skills score were observed at day-3 compared to pre-tests score in group A who received *HistoRM* game intervention first (p < 0.001) as well as in group B who received practicum handouts first (p = 0.014). Similarly, cognitive skills scores were observed at day-7 compared to pre-tests (p < 0.001). Day-7 post-tests were carried out to determine whether the increase in cognitive skills score was related to the order of intervention, *HistoRM* game followed by practicum handouts (group A) or practicum handouts in the first three days, followed by *HistoRM* game (group B). It was found that the increases in cognitive skills scores were higher in group A (1.35-fold) than group B (1.28-fold) at day-3 (p = 0.003)and day-7 post-tests (p = 0.02)(Fig. 4i-iv).

The positive impact of *HistoRM* game on student understanding of topic 2 (histology of tooth and its supporting structure) was also observed (p < 0.001). However, it was found that the increase in cognitive skills scores were higher in group A than group B at day-3 (p = 0.039) and not significantly different at day-7 post-tests (p = 0.06). Although the intervention of group A and B was changed (crossover). The data indicated the order of intervention did not influence the results. Furthermore, the improvement of cognitive skills scores was influenced by the number of missions completed. Pearson correlation analysis revealed positive correlation of students who completed the 15 missions have a higher day-7 post-tests score (p = 0.03).

### Student perceptions of HistoRM game as complementary learning of oral histology

In the learning content domain, students responded positively to the content of *HistoRM* game. Over 95% of participants reported that they gained a better understanding of histology content. More than 90% of students perceived the quiz sessions in *HistoRM* game helped them to understand the oral histology practicum lessons. They agreed (81%) that the content of *HistoRM* game has been previously studied. The practicum course is integrated with the theory of oral biology course and serves as the reinforcement of the theory class. In the *HistoRM* game domains, all of the questions received more than 90% positive responses, although not all students like to play games in their spare time for entertainment purposes. In the learning experience domain, the immediate feedback given after each quiz session helped the students understand the subject matters (98.3%) and agreed that serious games can be implemented as a complementary learning method. They suggested the serious games approach is not only used for learning oral histology but for other learning materials (Table 3).

**Table 3:** Student Perception of Serious Games as Complementary Learning of Oral Histology

Statements	N (%)				Mean Preference ± SD
	Strongly Disagree	Disagree	Agree	Strongly Agree	
A. Learning Content Domain					1.676 ± 0.626
1. Histologic images was clear	0 (0%)	1 (1.4%)	38 (51%)	35 (47.6%)	
2. Educational content in the game has been studied before	1 (1.4%)	13 (17.6%)	50 (67.5%)	10 (13.5%)	
3. The questions in the games helped me understand the practicum learning material	0 (0%)	2 (2.7%)	27 (36.5%)	45 (60.8%)	
B. HistoRM Games Domain					1.662 ± 0.625
4. Gameplay was fun	0 (0%)	6 (9%)	36 (49%)	31 (42%)	
5. Gameplay was easy	1 (1,4%)	6 (9%)	42 (57%)	25 (32,6%)	
6. Game design is good (including visual elements such as images, illustration, animation, colors, and layout)	0 (0%)	0 (0%)	37 (50%)	37 (50%)	
7. Text for questions and instructions were clear (size and number of text)	0 (0%)	1 (1.4%)	35 (47.6%)	38 (51%)	
8. Language use was easily understandable	0 (0%)	0 (0%)	34 (46.2%)	39 (53.8%)	
C. Learning experience Domain					1.601 ± 0.620
9. The feedback helped me understand the practicum learning material	0 (0%)	2 (2.7%)	29 (40.3%)	42 (57%)	
10. Games based learning method can be developed as a complementary learning method	1 (1.4%)	3 (4.8%)	35 (47.6%)	34 (46.2%)	
11. I have a hobby of playing electronic games for entertainment purpose	0 (0%)	23 (31.6%)	32 (43.7%)	18 (24.7%)	
12. The games help me to recall the previously learned subject materials	0 (0%)	1 (1,4%)	27 (36,5%)	46 (62,1%)	
13. Serious games as learning strategy helped me to understand learning materials of oral histology	0 (0%)	3 (4,8%)	27 (36,5%)	43 (58,7%)	
14. The allocated time to play the games was sufficient	0 (0%)	9 (12.2%)	38 (51.0%)	27 (36.8%)	

## Discussion

The present study was conducted to evaluate new learning strategies for interactive learning environments with asynchronous settings in dental education, in particular, the study of oral histology. The results of this study showed *HistoRM* as complementary learning of oral histology could improve students’ understanding of the content of knowledge acquisition, increase student interest to learn dental basic science and increase their learning motivation. The data demonstrated *HistoRM* game significantly improved students cognitive skills of oral histology compared to practicum handouts script-based group. The improvement was related to the duration to play the *HistoRM* and the number of missions completed. Student perception toward *HistoRM* game to increase their understanding of the microscopic structure of oral tissues was positive. They acknowledged the *HistoRM* game provided a more interesting learning environment. The data was in agreement with other studies on serious games in various health sciences that demonstrated an increase in learning satisfaction and positive attitude of students who use serious games as a learning medium [22–31]. The improvement in cognitive skills can be attributed to several game characteristics that encourage learning and changes in attitude and behavior, such as the presence of goal setting, decision making, problem solving, social rewards, and the use of stories in games that incorporated the concept of behavior change [25]. Moreover, strong engagement in learning has been reported related to academic achievement [32].

The main consideration for combining games with education is the ability of games to provide high motivation and potentially make the learning process easier, especially for the learning subjects that students perceive as less interesting. Serious games are different from traditional learning and are considered to provide a more interactive learning environment, support increased motivation, and safe training for students [33–36]. This occurs because of the different characteristics between conventional learning and games. Conventional learning emphasizes the transmission of information and memory. Meanwhile, games confront players with engaging problems and offer ways to explore the problems [8]. In this way, players have the opportunity to develop a higher level of learning such as analysis and evaluation. Analysis of serious games explains how student competence can be increased with formative feedback until the game is over [14]. In *HistoRM*, we provided direct feedback, to allow students to identify the results, to recognize mistakes based on previous experiences and reconsider strategies to complete the game. Students can improve their competence from the feedback provided by the game system to complete the games. Immediate feedback on serious games encourages users to learn from experience and is perceived by students as one of the strong features in the *HistoRM*.

The application of challenges in serious games can make learning activities more interesting and motivate students to complete the games. However, there must be a balance between the challenge and the student’s ability. If the challenge is too difficult to complete, the students could lose motivation to complete the game [15]. On the other hand, if the game is too simple, students may be bored and stop playing the game as well. This argument could be explained by the flow theory [37]. Good game design creates appealing experiences for players that integrate the engagement with educational effectiveness. In *HistoRM*, the game tasks were designed to be challenging, however we provided a hint feature that became available when students could not complete the mission at the designated time frame. In the process of solving challenges, the learning process occurs when students build their own concepts when they fail. Errors or failures in the games could improve the competence and skills of students in completing the games. Furthermore, the entertainment element is also needed to maintain the user’s attention in each game cycle, to avoid users who stop playing before successfully completing the game and do not understand the intended learning outcomes [8, 14]. An interesting entertainment component is also important and has an impact on knowledge acquisition. The use of video games, interactive music, high-quality graphics, color combinations, animation, and presentation of material is intended to increase visualization, sensations of interactivity, motivation, interest, player involvement in the game cycle and the impact on knowledge acquisition [38]. Game cycle is an iterative learning process that involves user assessment, user behavior, and a feedback system which, with repetition or repetition of cycles, can lead to the achievement of learning outcomes [14, 15]. Games need to be created interesting enough so that players are interested and motivated to repeat the game cycle. A game cycle can be triggered by two components, namely a combination of learning content with game characteristics so that students are stimulated to learn [8].

The present study highlights the importance of introducing an innovative learning approach to enhance knowledge acquisition and improve the overall learning environment. However, this study had some limitations. The first limitation of the study was the relatively low response rate of 53.6% of total first year dental students, although students were strongly encouraged to take part in this study, their participation remained voluntary. The number of non-respondents might undermine the power of the study and therefore the potential response bias can not be completely ruled out. The results of the study must be interpreted with caution. Second, the generalizability of the results of the present study was limited by the use of data from a single university with a discrete population of first year dental students. As each dental school may have different conditions that influence the use of serious games in their curriculum [31], the results of the study must therefore be interpreted with caution. Further studies are needed to explore serious games as learning approaches in other settings. Third, future cohort studies evaluating the impact of *HistoRM* in knowledge retention are also crucial to measure long-term benefit to the learning method. Finally, as social interactions can have an impact on how students could gain knowledge from game-based learning [29], future research should also investigate whether collaborative or competitive approaches could be supportive for the game.

## Conclusion

Based on the results of the present study, it can be concluded that *HistoRM* serious game effectively improved students’ understanding of oral histology learning outcomes and provided more interesting learning experiences. This innovative learning tool can be recommended as a complementary learning strategy of oral histology for dental students.

### Electronic supplementary material

Below is the link to the electronic supplementary material.

Supplementary Material 1

Supplementary Material 2

## Data Availability

All datasets used and analyzed in this study will be available from the corresponding author on reasonable request.

## Abbreviations

ICC: Inter correlation coefficient
