# Supplementary material for: Serious game as oral histology learning strategy for undergraduate dental students; crossover randomized controlled trial
Source: BMC Oral Health. 2023 Aug 23;23:585. doi: 10.1186/s12903-023-03286-3 (PMC10463426; doi:10.1186/s12903-023-03286-3)
Supplement: Supplementary file 2 — Supplementary Material 2 [file 12903_2023_3286_MOESM2_ESM.pdf]

Questions	Strongly disagree	Disagree	Agree	Strongly agree
Serious Game ID				
1. Histologic images was clear				
2. Educational content in the game has been studied before				
3. The questions in the games helped me understand the practicum learning material				
4. Gameplay was fun				
5. Gameplay was easy				
6. Game design is good (including visual elements such as images, illustration, animation, colors, and layout)				
7. Text for questions and instructions were clear (size and number of text)				
8. Language use was easily understandable				
9. The feedback helped me understand the practicum learning material				
10. Games based learning method can be developed as a complementary learning method				
11. I have a hobby of playing electronic games for entertainment purpose				
12. The games help me to recall the previously learned subject materials				
13. Serious games as learning strategy helped me to understand learning materials of oral histology				
14. The allocated time to play the games was sufficient				
